# Optimization of Case Definitions for Sensitivity as a Preventive Strategy—A Modelling Exemplified with Rapid Diagnostic Test-Based Prevention of Sexual HIV Transmission

**DOI:** 10.3390/diagnostics11112079

**Published:** 2021-11-10

**Authors:** Andreas Hahn, Hagen Frickmann, Ulrike Loderstädt

**Affiliations:** 1 Institute for Medical Microbiology, Virology and Hygiene, University Medicine Rostock, 18057 Rostock, Germany; andreas.hahn@uni-rostock.de (A.H.); frickmann@bnitm.de (H.F.); 2 Department of Microbiology and Hospital Hygiene, Bundeswehr Hospital Hamburg, 20359 Hamburg, Germany; 3 Department of Hospital Hygiene & Infectious Diseases, University Medicine Göttingen, 37075 Göttingen, Germany

**Keywords:** rapid diagnostic testing, RDT, sensitivity, modelling, symptoms, transmission prevention, infectious disease, human immunodeficiency virus, HIV

## Abstract

In clinical studies, case definitions are usually designed to optimally match the desired clinical state, because lacking specificity is associated with a risk of bias regarding the study outcome. In preventive medicine, however, high sensitivity is sometimes considered as more critical in order not to overlook infectious individuals, because the latter may be associated with ongoing spread of a transmittable disease. Accordingly, this work was focused on a theoretical model on how the sensitivity of case definitions can be optimized by adding clinical symptoms to diagnostic results for preventive purposes, if the associated reduction in specificity is considered as acceptable. The model was exemplified with an analysis on whether and in how far exposure risk can be reduced by the inclusion of observable symptoms during seroconversion syndrome in case of rapid diagnostic test-based prevention of sexual HIV transmission. The approach provided a high level of safety (negative predictive values close to 1) for the price of a considerably number of false positives (positive predictive values < 0.01 for some subpopulations). When applying such a sensitivity-optimized screening as a “diagnostics as prevention” strategy, the advantages of excellent negative predictive values need to be cautiously balanced against potential undesirable consequences of low positive predictive values.

## 1. Introduction

As recently demonstrated by our group, imperfect accuracy both of diagnostic results [1] and of case definitions [2] can interfere with the outcome of clinical trials in an undesirable way. Accordingly, it is advisable to optimize case definitions for specificity in the most study contexts in order to reduce respective sources of bias [2]. If this is not feasible, sensitivity and specificity of both diagnostic assays [1] and case definitions [2] should at least be known, so diagnostic accuracy-adjusted estimators [3,4] can be applied in order to reduce the effects of associated bias on the study outcomes.

Although optimization of case definitions for specificity may be appropriate for the most instances, however, this does not necessarily apply in all situations. The costs for optimized specificity usually include acceptance of reduced sensitivity [5], implying that a few “cases” may go undetected if highly specific case definitions are applied.

Although both case definitions and diagnostic tests usually try to come as close as possible to the abstract “unknown” truth, “perfect” accuracy for both of them is usually not to be expected in a real-world setting [6]. Because, however, optimization of specificity can usually only be achieved for the price of reduced sensitivity and vice versa [5,6,7], medical, scientific and even political decision makers will necessarily have to balance potential beneficial and negative consequences of such optimization in the one or the other direction. Abstractly spoken, science may help to quantify the effects of such decisions, but the decision itself within such a balancing will stay a normative one and will depend on the aims of the decision maker.

Optimization for sensitivity rather than for specificity may, e.g., be of interest for public health decision makers in situations when infectious individuals shall not go undetected in order to prevent the further spread of an infectious disease. This is particularly the case, if the consequence for individuals in case of false positive results are mild and can be easily corrected, while severe medical consequences may result from the spread of an infectious disease. Under such circumstances, preventive medical purposes may facilitate balancing more in the direction of optimized sensitivity rather than in the direction of optimized specificity, if the benefits arising from the prevention of the spread of an infectious disease are considered as relevantly more important than potential negative consequences arising from false positive results.

In the exemplarily modelling described here, we introduce how—by themselves—non-specific observable symptoms may contribute to an increased sensitivity of a case definition which would otherwise rely on a diagnostic assay with imperfect sensitivity alone. Based on the abstract model as presented in the Materials and Methods section, exemplification is conducted with the example of the inclusion of seroconversion syndrome-associated observable symptoms in rapid diagnostic test-based prevention of sexual HIV exposition. Associated advantages and disadvantages are discussed in order to demonstrate potential chances and risks of the abstract concept of sensitivity-optimized case definitions for public health interventions. For this purpose, the HIV pandemic was just exemplarily chosen, because 40 years of experience with the HIV pandemic resulted in the availability of epidemiological details which facilitate modelling approaches based on well-defined epidemiological evidence.

## 2. Materials and Methods

### 2.1. The Mathematical Background Underlying the Exemplary Modelling

A sensitive case definition for the identification of an infection may not only include a positive result of a diagnostic test but also a couple of symptoms that have been identified as being associated with this infection. Such a case definition would be fulfilled if the diagnostic test was positive or one or more of the respected symptoms were observed. Based on those assumptions, the overall sensitivity of such case definition is given by:

$$\mathrm{Sensitivity}=1-\left(1-{\mathrm{Sensitivity}}_{\mathrm{Diagnostic}\;\mathrm{Test}}\right)\times \left(1-{\mathrm{Sensitivity}}_{\mathrm{Symptoms}}\right)$$

The specificity is given by:

$$\mathrm{Specificity}={\mathrm{Specificity}}_{\mathrm{Diagnostic}\;\mathrm{Test}}\times {\mathrm{Specificity}}_{\mathrm{Symptoms}}$$

When such a case definition is applied to prevent exposition events towards infections, its positive and negative predictive values (*PPV*, *NPV*) are essential to evaluate its performance. The predictive values *PPV* and *NPV* are given by:

$$PPV=\frac{Sensitivity\times Prevalence}{Sensitivity\times Prevalence+\left(1-Specificity\right)\times \left(1-Prevalence\right)}$$

$$NPV=\frac{Specificity\times \left(1-Prevalence\right)}{Specificity\times \left(1-Prevalence\right)+\left(1-Sensitivity\right)\times Prevalence}\;$$

With the reciprocal of *PPV* and *NPV*, the number of positive test results needed to get a true positive result and the number of negative test results needed to get a true negative test result are defined, respectively.

With focus on the equation for the *PPV*, it is immediately evident that in case of low prevalence, even perfect sensitivity is not necessarily associated with a good positive predictive value. This is only the case if specificity is almost ideal. Even small deviations from this optimum can lead to a collapse of the positive predictive value. If it is not intended to maximise the sensitivity of the case definition without regard to the positive predictive value, care should be taken in the construction of the case definition to ensure sufficient specificity so that the positive predictive value does not fall below a minimum that is considered as acceptable. The minimum specificity required for a desired sensitivity, prevalence and the minimum positive predictive value still considered as acceptable are given by:

$$Specificity=1-\frac{Sensitivity\times Prevalence\times \left(1-PPV\right)}{PPV\times \left(1-Prevalence\right)}$$

The sensitivity and the specificity of the symptoms partly depend on the number of independently distributed symptoms that shall be observed to fulfill the symptoms-related component of the case definition. If there are n symptoms and 1 ≤k≤n of them have to occur that a patient is “symptomatic” in line with the case definition, then sensitivity and specificity of the symptoms-related component of the case definition are given by:

$$\begin{array}{cc} Sensitivity= & P(\geq k\;symptoms\;occur|\;individual\;is\;infected)\\ & \;\;\;=1-P\left(<k\;symptoms\;occur|\;individual\;is\;infected\right) \end{array}$$

Specificity=P(<k symptoms occur | individual is not infected)

It should be noted here that the probability of the occurrence of the symptoms may differ and that they are therefore Poisson binomially distributed.

### 2.2. Assumptions and Prerequisites for the Example of Rapid Diagnostic Test-Based Prevention of Sexual Exposure towards HIV

#### 2.2.1. Summary of the Testing as Prevention Concept with Focus on HIV

As recently demonstrated by our group based on three previous modelling approaches [8,9,10] and summarized in a mini-review [11], a combination of self-testing and the testing of potential sexual partners applying traditional or molecular rapid diagnostic testing (RDT) strategies can be a promising approach for the transmission prevention of sexually transmitted infections (STIs) for individuals who do not want to use condoms. As discussed previously [8,9,10,11,12], the effectiveness of such test-based preventive strategies depends on various factors, including the availability of reliable and easy-to-apply diagnostic point-of-care-testing (POCT) solutions even for diagnostic laymen, window-periods of the applied tests as well as the tests’ sensitivity and specificity.

In Germany, purchasing of RDTs targeting infections with the human immunodeficiency virus (HIV) by laymen is legally possible since June 2018 [13] as an element of the national strategy for the prevention of HIV transmission. Although self-testing is the intended use of such RDTs, it is nevertheless technically simple to use them for reciprocal testing among potential casual sexual partners who are interested in proving each other “mutually assured” HIV negativity prior to engaging in sexual activity without condom protection. In case of intercourse with sex workers, such condom-free sex is prohibited in Germany since July 2017 by § 32 of the Sex Worker Protection Act (“Prostitutionsschutzgesetz”), demanding condom use in case of all commercial sexual contacts. As sex workers, however, have initially invented the abovementioned “diagnostics as prevention” strategy to protect themselves against HIV transmission in case of agreed unprotected sexual intercourse with their clients long before even the purchasing of HIV RDTs was legally possible [9], it is likely that they will illegally proceed with this strategy in the demimonde. Next to commercial sex work, casual sexual encounters as well among risk groups with high HIV prevalence such as men having sex with men (MSM) may represent situations wherein individuals are potentially interested in reciprocal HIV testing applying RDTs [8,9].

In the first year of implementation of freely availably HIV RDTs in Germany, an estimated quantity of 30,000 tests have been sold and applied [14]. The German society for the support of patients with acquired immunodeficiency syndrome (AIDS) (“Deutsche Aids-Hilfe”) considers the strategy of making HIV RDTs freely available for self-testing purposes as a success in the struggle against the ongoing HIV pandemic [15].

Regarding the “diagnostics as prevention” strategy of reciprocal HIV testing by potential sex partners, however, a window-period of traditional immunochromatographic RDTs limits the reliability of this preventive strategy during acute HIV infection, also called seroconversion stage [9]. For this stage of the HIV infection, which is characterized by high viral loads with associated high transmission risk but antibody levels yet below the detection threshold [8,15,16,17,18,19,20], a combination of molecular RDTs based on polymerase chain reaction (PCR) or loop-mediated isothermal amplification (LAMP) in addition to traditional immunochromatography would be desirable as recently shown to further reduce both the HIV exposition and transmission risk [8,9]. Beyond well-equipped hedonistic clubs, however, availability of molecular HIV testing is presently hardly realistic in the most contexts of risky casual sexual contacts.

#### 2.2.2. Concept of the Inclusion of Seroconversion-Related Symptoms to Increase Sensitivity of the Testing as Prevention Approach

To circumvent the problem of low sensitivity of immunochromatographic RDT-based HIV testing during the seroconversion stage, individuals with affinity to condom-free sex might increase the sensitivity by including symptoms which occur in defined percentages in the course of acute HIV infection/seroconversion syndrome [21,22] in the case definition. Such symptoms could be assessed by direct questioning, but there are reasons which speak against this option. Firstly, medical questioning in a situation of erotic adherence might pose a social challenge. Secondly, as known from strategies trying to avoid reporting bias in studies on sexual medicine [23,24,25,26,27], truthful reports in the context of sexual issues cannot regularly be expected. This could be particularly the case if truthful statements might lead to exclusion from the desired sexual activity. Accordingly, it will be useful to include only symptoms that can be directly checked and verified by the potential sexual partner, before a final decision for or against condom-free sex is made.

As the symptoms of HIV–seroconversion are not specific to acute HIV infections, their inclusion will necessarily lead to a tremendous decrease in specificity of the case definition compared to a case definition based on a positive RDT alone. However, if the consequence of a false positive result is just the use of condoms instead of unprotected sex, prioritizing of sensitivity over specificity may be acceptable in comparison to a slightly higher risk of HIV infection [9]. While in the context of the most studies, optimization for specificity is desirable [1,2], the example provides a situation in which optimization of the case definition for sensitivity seems appropriate. Thereby, the inclusion of directly verifiable disease-associated symptoms into a case definition may help to increase the sensitivity of RDTs, a decision which has to be weighted against lower specificity.

In particular, a case definition for the identification of acute HIV infection (seroconversion syndrome) may include clinical symptoms that occur at an early stage of infection when RDT testing still shows a lack of sensitivity. For the modulation, it has to be assumed that these symptoms are independently distributed. In Table 1, common symptoms of an acute HIV infection with known likelihood of occurrence are given as previously reported [21]. Focussing on symptoms that are sensorially (visibly, tactilely, etc.) verifiable by a third person, such symptom-based case definitions will define an individuum as “positive” in line with the symptom-based case definition if there is at least one of the included symptoms present. Accordingly, the case definition will not be fulfilled if none of the symptoms occurs as chosen for the case definition in Table 1.

The sensitivity of the symptom-related proportion of a case definition based on the included symptoms from Table 1, assuming that at least one of the independently distributed symptoms exists, is given by:

$$Sensitivity=1-\overset{n}{\underset{i=1}{\prod }}\left(1-p_{i}\right)$$

Thereby, *p_i_* is the likelihood of symptom occurrence in the course of acute HIV infection. Assuming the likelihoods in Table 1 for the included symptoms, the sensitivity of this case definition is 0.94.

The specificity of this case definition strictly depends on the distribution of the symptoms among the non-infected population. Thereby, “non-infected” means that an individuum is not in an acute stage of HIV infection. Accordingly, the specificity of the case definition is the likelihood that none of the included symptoms from Table 1 occurs in the non-infected population and is given by:

$$Specificity=\overset{n}{\underset{i=1}{\prod }}(1-p_{i})$$

Thereby, (1 − *p_i_*) is the likelihood that a symptom *i* will not occur within the non-infected population.

Since there are no reliable information on the distribution of the most of those more-or-less non-specific symptoms among the non-infected population, the model was adapted to the following different assumptions of symptom distribution among the non-infected individuals: Assuming possible likelihoods that at least one of the chosen symptoms occurs in a non-infected individual are given by 0.01%, 0.1%, 1%, and 10%, the resulting specificity of the case definition is given by 0.9999, 0.999, 0.99, and 0.9, respectively.

When a case definition as given above is applied to prevent sexually transmitted HIV infections, its positive and negative predictive values (*PPV*, *NPV*) are essential to evaluate its performance.

#### 2.2.3. Assumption Regarding Prevalence and Incidence of HIV Infections as Well as Description of the Stages of HIV Infection as Applied for the Modelling

For Germany, prevalence and incidence of HIV infection for the exemplarily chosen pre-pandemic year 2015 (without differentiation between acute and non-acute HIV infection) are given in Table 2.

The frequency of acute HIV infections can be estimated based of the cumulative duration of each stage of the HIV infection as given in Table 3.

Based on a lack of sensitivity of the RDT chosen for the modelling [9] in the first month of a HIV infection, the incidence of HIV was weighted by the factor 31/365. This assumption results in a weighted incidence of 31.0 females, 31.9 heterosexual males, and 186.9 men who have sex with men (MSM) for the year 2015.

For the diagnostic performance of the assessed Ab/Ag RDT [9,12], the following diagnostic sensitivity and diagnostic specificity after day 10 for the antigen component of the RDT and after day 31 for the antibody component of the RDT were assumed as described elsewhere [9]:

- Antibody component: sensitivity 0.973 and specificity 0.996 (applicable from day 32 after infection);
- Antigen component: sensitivity 0.123 and specificity 0.997 (applicable from day 11 to day 31 after infection).

For the combination of the symptom component of the case definition *S* and the RDT component of the case definition, positive and negative predictive values *PPV_C_* and *NPV_C_* are given by:

$$PPV_{c}=\frac{\left(Se_{S}+Se_{RDT}-Se_{S}\times Se_{RDT}\right)\times Prev_{HIV}}{S+RDT-S\times RDT}$$

$$NPV_{C}=\frac{NPV_{S}\times NPV_{RDT}\times \left(1-S\right)\times \left(1-RDT\right)}{\left(1-S\right)\times \left(1-RDT\right)}=NPV_{S}\times NPV_{RDT}$$

Thereby, *S* and *RDT* indicate the expected value of a positive result of the symptom-based case definition or the RDT-based case definition, respectively.

Further, it is assumed that the distribution of the symptoms representing the symptom-based case definition among HIV infected individuals after day 31 of infection is comparable to the distribution of seroconversion-like symptoms among non-HIV-infected individuals, because the seroconversion stage is close to its end or over. In addition, it is taken for granted that the symptom-based case definition and the RDT-based case definition are stochastically independent.

## 3. Results

### Exemplary Modelling of a Sensitivity-Optimized Case Definition Combining Rapid Diagnostic Test Results with Seroconversion-Associated Symptoms for the Prevention of Sexual HIV Exposition

Based on the assumptions above, the symptoms component of the case definition for the identification of an acute HIV infection results in very low positive predictive values for females and heterosexual males. Its application in the MSM community alone is associated with acceptable positive predictive values if the prevalence rate of the symptoms defining the case definition is very low among the non-infected individuals. The latter means that the prevalence rate of occurrence of at least one of the symptoms in the non-infected population is 0.001 or lower. In this case, the likelihood that a positive result is correct can be expected to be 0.6843. In other words, 1.47 individuals have to fulfill this element of the case-definition in this situation to get one correctly positive test result. In females and heterosexual males, the positive predictive value of such a case definition is almost zero. On the other hand, the negative predictive values are equal to one over all populations and thus, they are also identical with the pretest probability (Table 4).

Additionally, for stages of HIV infection after day 10, the positive predictive values for Ag/Ab RDT-based case definitions are very low in the female and heterosexual male population but much higher than in the scenario for newly infected individuals. Especially for the MSM population, positive and negative predictive values of the RDT-based approach are very high (Table 5). As shown for the symptom-based approach above, the negative predictive values are high over all populations.

Although the sensitivity of the symptom-related component of the case definition is 0.94 until day 31, the weighted sensitivity over all stages of HIV infection reduces it to 0.2% for females and heterosexual males and to 0.3% for the MSM population if it is interpreted as a diagnostic test for HIV infection in general and if it is assumed that the distribution of seroconversion-like symptoms of this case definition among the HIV infected population after day 31 is the same as among non-infected individuals. The diagnostic sensitivity of the antigen-component of the RDT (Ag) as a diagnostic test weighted over all stages of HIV infection is reduced to 0 while its specificity is increased to one. For the antibody-RDT-component (Ab), sensitivity is slightly reduced while the specificity is slightly increased (sensitivity and specificity of 0.971 and 0.998 in females and heterosexual males, respectively, as well as 0.970 and 0.999 in the MSM population, respectively).

Combining the symptom-related component of the case definition with the RDT component of the case definition as a diagnostic test for HIV in general provides higher positive predictive values than separately assessed elements of the case definition but remains at a very low level among females and heterosexual males. Among the MSM population, the combined case definitions result in appropriate positive predictive values if the symptom distribution among the non-infected individuals is up to 1% or lower.

As the latter distribution of the symptoms of the symptom-based case definition is uncertain, the Ab/Ag-RDT can be proposed as the most reliable test strategy among the MSM population (Table 6).

## 4. Discussion

The modelling-based study presented here had a number of aims. Firstly, a model was designed for the increase in sensitivity of case definitions by compensating for the limited sensitivity of a diagnostic test in the early stage of a disease by the inclusion of known symptoms of the respective disease stage. The idea was that such a model might be useful for RDT-based exposition prevention in a pandemic, a concept which has been widely used for the management of the SARS-CoV-2 (severe acute respiratory syndrome-coronavirus 2) pandemic [28] and mostly applying rapid-diagnostic tests with imperfect diagnostic accuracy [29,30,31]. Accordingly, case definitions in our modelling were not optimized for specificity [1,2], as it is usually desirable in case of clinical trials, but for sensitivity, as the sole aim was the reduction in the exposition risk.

Secondly, the model was tested with a specific example. Due to longer experience with the respective pandemic and thus higher reliability of available datasets for the modelling, the model was not exemplified with the SARS-CoV-2 pandemic but with the HIV-pandemic [32].

As expected, optimization of the case definition for sensitivity had both beneficial and undesirable effects. Based on the known likelihood of defined objectifiable symptoms of HIV seroconversion syndrome [21,22] and the test characteristics of a common HIV RDT targeting both gp24 antigen and HIV-specific antibodies as extracted from a meta-analysis [12], an increase in sensitivity of the case definition “potential HIV seroconversion syndrome” was observed from 12% in case of sole reliance on the RDT results to 94% if objectifiable and verifiable symptoms were included. Thereby, it is of course debatable whether or not the included symptoms are really “recognizable” for medical laymen without respective diagnostic experience, so the practical effect will most likely be lower than the hypothetical one.

Lacking reliable data on the common distribution of the included non-disease-specific, usually mild symptoms in the non-HIV-infected population made an assessment of the specificity of the combined case definition impossible, so only assumptions could be made. Due to the lacking disease-specificity of the included symptoms, however, it has to be assumed that the specificity of such a case definition will be very low, which is a major and expected disadvantage of the approach.

More than this, when applied, for example, to the German “standard” population with an extremely low number of incidental people in the very early stages of HIV infection, even the uncertainty regarding the exact specificity value is practically hardly relevant for the resulting predictive values: The negative predictive value is virtually always close to 1, the positive predictive value is always virtually 0 due to the extremely low number of infected people in the early phase by applying time-weighted incidence for the calculations. Accordingly, the practical information gained when using such a combined case definition, i.e., its reliability for the diagnosis of the HIV seroconversion syndrome, is practical zero with focus on both the positive as well as the negative predictive value.

This, however, does not apply to the exposure risk. In spite of poor predictability of HIV seroconversion syndrome, the exposure probability could be reduced if the case definition was applied correctly. Whereby, however, it would be accepted that the proportion of false positives was of course enormous. If the consequence of this relevant limitation is just a switch from non-protected to protected sexual intercourse; however, the extremely low positive predictive value may be considered acceptable for potential sexual partners willing to protect themselves by reciprocal testing.

With focus on the quantitative dimension of risk reduction in case of the HIV–seroconversion example, it can be concluded that the case definition amended by non-specific symptoms does not offer a relevant increase in safety, as the initial pretest probability of HIV seroconversion is simply too low within the average German population. For high-risk populations including men having sex with men, the risk reduction is slightly better, making such an approach with an extremely high sensitivity potentially useful. So, in case of doubt in high-risk communities, a respective high-sensitivity-case-definition might be considered. However, the effect on exposure risk reduction in addition to RDT testing alone will be within the homeopathic range, in particular in case of heterosexual contacts, and only slightly better in the MSM setting.

As exemplified with the HIV pandemic, optimization of case definitions for sensitivity by adding non-specific clinical symptoms [21,22] even to highly specific diagnostic tests [12] can have deleterious consequences on the predictive values. This particularly applies in case of low prevalence of the assessed medical condition and, accordingly, a resulting low pre-test probability. The professional decision on whether or not such an approach may nevertheless be acceptable in a pandemic will largely depend on the expected medical consequences in case of a transmission event.

In the abovementioned example, the still considerable medical consequences of acquiring an HIV infection in terms of requirement for lifelong medical treatment may be balanced against the minor inconvenience of switching from non-protected to protected sexual intercourse. So, the consequences of the high likelihood of false positive results may be considered as acceptable by individuals applying such a sensitivity-optimized “diagnostics as prevention”-based approach of reciprocal RDT-based HIV-testing.

If, however, medical or social consequences of a false positive result are more severe, e.g., defining a need for long isolation periods or quarantine periods for contact persons in a pandemic caused by pathogens other than HIV, the ethical balancing will become more complex.

## 5. Conclusions

As demonstrated by the model and the example, sensitivity of RDT-based diagnosis in pandemic situations can be considerably increased if non-specific clinical symptoms are included. In particular in case of low prevalence of the diagnosed infectious disease and thus poor pre-test probability, however, the predictive values can be tremendously deteriorated, but the exposure prevention effect can still be increased. Thereby, it has to be decided—balancing both the medical consequences of a transmission event and the social consequences of a false positive result—whether the associated high probability of false positive results in case of applying such case definitions appears justified or not.

The presented modelling has a number of implications for public health decisions in the course of a pandemic. The inclusion of symptoms in case definitions is of particular interest when the former can be clearly identified without the need for medically trained personnel, so that simple mass application seems realistic. The use of such case definitions including symptoms appears to be particularly useful when infectivity is already present before diagnostic detectability, for example by means of rapid tests, because symptoms have already developed to some extent within the diagnostic window period, as illustrated by the example of HIV. However, if the occurrence of symptoms and the detectability of the disease by test assays with poor assay specificity coincide, it seems reasonable to change the linkage of these two components of the case definition from “or” to “and”, because an increase in specificity is then advisable. The verification of the suitability of such a case definition optimized with respect to specificity instead of sensitivity is still pending and should be investigated in future studies.

## Figures and Tables

**Table 1:** Proportion of common symptoms of acute HIV infection/seroconversion stage according to [21]. Only symptoms sensorially verifiable by a third person were included in the case definition (right column).

Symptom	P (Symptom|Acute HIV)	Chosen for the Case Definition
Fever	0.746	x
Fatigue	0.677	
Myalgia	0.484	
Skin rash	0.479	x
Headache	0.450	
Pharyngitis	0.405	
Cervical adenopathy	0.389	x
Night sweats	0.296	
Arthralgia	0.278	
Diarrhoea	0.267	
Inguinal adenopathy	0.196	x
Weight loss	0.150	
Stomatitis	0.045	x
Oral/oesophageal candidiasis	0.021	x
Unilateral or bilateral tonsillitis	0.003	
Severe gastritis	0.003	
CMV gastritis and/or colitis	0.003	
Acalculous cholecystitis	0.003	
Bell’s Palsy and/or Paresis	0.01	x
Acute psychiatric disorder	0.01	x
Encephalitis	0.007	
Multi-segmental herpes zoster	0.003	x
Peripheral polyradiculoneuritis	0.003	
Distal paraesthesia, aphasia	0.003	
Pneumonia and/or URTI	0.017	
Hair loss	0.007	

P = probability.

**Table 2:** Prevalence and incidence of HIV in Germany in 2015 as described by the Robert Koch Institute, i.e., the central institution of the German federal government with responsibility for disease monitoring and prevention.

	Females Absolute/Frequency	HET Males Absolute/Frequency	MSM Absolute/Frequency
Prevalence	15,200/3.6 × 10^−4^	13,362/3.4 × 10^−4^	56,138/6.9 × 10^−2^
Incidence	365/8.8 × 10^−6^	375/9.4 × 10^−6^	2200/2.7 × 10^−3^
Population size	41,661,600	40,514,100	810,282

HET = heterosexual. MSM = men having sex with men.

**Table 3:** Viral load by stage of infection according to [18,19,20] as summarized by our group in [8].

Stage of Infection	Viral Load (Median Copies/mL)	Viral Load (Median log_10_)	Individual Duration in Days	Cumulative Duration in Days
Stage 1	2110	3.32	5.0	5.0
Stage 2	258,229	5.41	5.3	10.3
Stage 3	259,465	5.41	3.2	13.5
Stage 4	170,000	5.23	5.6	19.1
Stage 5	18,700	4.27	69.5	88.6
Chronic stage (6)	31,623	4.5	Open-end	
Stage under successful treatment (7)	10	1	Open-end	

**Table 4:** Positive and negative predictive values of the symptoms component of the case definition depending on various assumed specificity rates until day 31.

Population	Assumed Specificity of the Symptoms	Positive Predictive Value	Number Needed to Test Positive for a First Correctly Positive Tested	Negative Predictive Value	Number Needed to Test Negative for a First Correctly Negative Tested
Females	0.9999	0.0069	143.97	1	1
	0.999	0.0007	1430.70	1	1
	0.99	<0.0001	14,298.04	1	1
	0.9	<0.0001	142,971.38	1	1
HET	0.9999	0.0073	136.32	1	1
	0.999	0.0007	1354.25	1	1
	0.99	<0.0001	13,533.60	1	1
	0.9	<0.0001	135,325.96	1	1
MSM	0.9999	0.6843	1.46	1	1
	0.999	0.1782	5.61	1	1
	0.99	0.0212	47.12	1	1
	0.9	0.0022	462.23	1	1

HET = heterosexual males. MSM = men having sex with men.

**Table 5:** Positive and negative predictive values for the Ag/Ab RDT component of the case definition after day 10.

Population	Positive Predictive Value	Number Needed to Test Positive for a First Correctly Positive Tested	Negative Predictive Value	Number Needed to Test Negative for a First Correctly Negative Tested
Females	0.0814	12.29	1	1
HET	0.0741	13.49	1	1
MSM	0.9475	1.06	0.9980	1

HET = heterosexual males. MSM = men having sex with men.

**Table 6:** Positive and negative predictive values for a combined case definition including both symptoms and Ag/Ab RDT results.

Population	Assumed Specificity of the Symptoms	Positive Predictive Value	Number Needed to Test Positive for a First Correctly Positive Tested	Negative Predictive Value	Number Needed to Test Negative for a First Correctly Negative Tested
Females	0.9999	0.1443	6.93	1	1
	0.999	0.1057	9.46	1	1
	0.99	0.0287	34.84	1	1
	0.9	0.0035	285.71	1	1
HET	0.9999	0.1323	7.56	1	1
	0.999	0.0965	10.36	1	1
	0.99	0.0261	38.31	1	1
	0.9	0.0031	322.58	1	1
MSM	0.9999	0.9823	1.02	1	1
	0.999	0.9712	1.03	1	1
	0.99	0.8728	1.15	1	1
	0.9	0.4334	2.31	1	1

HET = heterosexual males. MSM = men having sex with men.

## Data Availability

All relevant data are provided in the manuscript and its tables.
